# MIrExpress: A Database for Gene Coexpression Correlation in Immune Cells Based on Mutual Information and Pearson Correlation

**DOI:** 10.1155/2015/140819

**Published:** 2015-12-03

**Authors:** Luman Wang, Qiaochu Mo, Jianxin Wang

**Affiliations:** ^1^School of Information, Beijing Forestry University, Beijing 100083, China; ^2^Department of Natural Science in Medicine, Peking University Health Science Center, Beijing 100191, China; ^3^Center for Computational Biology, Beijing Forestry University, Beijing 100083, China

## Abstract

Most current gene coexpression databases support the analysis for linear correlation of gene pairs, but not nonlinear correlation of them, which hinders precisely evaluating the gene-gene coexpression strengths. Here, we report a new database, MIrExpress, which takes advantage of the information theory, as well as the Pearson linear correlation method, to measure the linear correlation, nonlinear correlation, and their hybrid of cell-specific gene coexpressions in immune cells. For a given gene pair or probe set pair input by web users, both mutual information (MI) and Pearson correlation coefficient (*r*) are calculated, and several corresponding values are reported to reflect their coexpression correlation nature, including MI and *r* values, their respective rank orderings, their rank comparison, and their hybrid correlation value. Furthermore, for a given gene, the top 10 most relevant genes to it are displayed with the MI, *r*, or their hybrid perspective, respectively. Currently, the database totally includes 16 human cell groups, involving 20,283 human genes. The expression data and the calculated correlation results from the database are interactively accessible on the web page and can be implemented for other related applications and researches.

## 1. Introduction

In recent years, the advance of microarray technology has provided amounts of information for us to observe the expression levels of genes together. Based on the increasing availability of gene expression data, public gene expression repositories were successfully constructed, such as the GEO [1] database and ArrayExpress [2]. These supply more opportunities to study gene expressional correlation (gene coexpression). Gene coexpression may reveal general functional tasks and regulatory mechanisms; moreover, it may identify novel genes to be involved in certain diseases. In addition, in the related fields of biology, many studies illustrated that the dependencies of gene expression can reflect the normal and dysfunctional biological processes and furthermore make us understand the underlying molecular mechanisms [3, 4]. It is difficult, however, for biologists without bioinformatics background to retrieve the gene coexpression information effectively and efficiently. For such, there in the field of plant biology are many coexpression databases, such as PLANEX [5], ATTED-II [6], Cop [7], TEGD [8], and PlantCART [9], the information in which was derived from large-scale gene expression data. Besides those, several coexpression databases peculiarly for mammals recently have been established and widely used by researchers and have thus accelerated the coexpression analysis process in the field of bioinformatics. COXPRESdb [10] was constructed with gene expression data from 63 human tissues and it utilizes the correlation rank to compare the coexpression strengths among multiple species. The database GeneFriends [11] adopted the same approach as COXPRESdb to construct coexpression maps based on transcriptome sequencing (RNA-seq) gene data instead of microarray gene data. HGCA [12] was constructed based on gene expression data from about two thousand samples of various cells and tissues. The overall correlation in gene expression was identified in this database across multiple tissues, or mixed tissues and cells, without meeting the necessity of coexpression in the same cell type. Immuco [13] is a cell-specific database in which gene expression values in each cell type across various conditions are provided, as well as gene coexpression and correlation information. Though these databases have been constructed successfully and are able to meet users' needs to some extent, they capture only the linear coexpression relationships between different genes by the Pearson correlation coefficient (value *r*). In fact, *r* with small absolute value of two genes does not necessarily mean that the two genes are independent, since nonlinear relationship may exist in the gene coexpression data [14]. In particular, two variables with a vanishing correlation coefficient may be heavily dependent, as illustrated in the later example in this paper (see Figure 2). The mutual information (MI) is able to measure the mutual dependence of two random variables, particularly in terms of positive, negative, and nonlinear correlations [15], and in comparison with Pearson correlation coefficient, it may provide a criterion better and more general to investigate gene coexpression. And in recent years, the mutual information is regarded as a common way to detect dependencies between different genes. Steuer et al. initiated the mutual information approach [16] for one specific gene dataset to analyze intergene dependencies.

Bioconductor is an open source software which provides the key function in Affymetrix array analysis in the R software environment (http://www.r-project.org/) [17], and Meyer et al. [18] developed a package “minet” in Bioconductor, in which a powerful tool is provided to calculate the mutual information between different gene pairs. Based on a publicly available dataset* Saccharomyces cerevisiae* [19] including 2,467 genes, Butte and Kohane applied the mutual information to measure gene-gene interaction and obtained the result that the mode of MI was about 0.7. Consequently, 22 relevance networks were constructed when the threshold of information (TMI) was set to 1.3 [20]. With gene expression data from various environments, the mutual information approach [21] was employed to reconstruct regulatory networks of relationships.

In spite of the many researches and applications mentioned above about mutual information for gene correlations, few publications related to mutual information focus on immune cells. Since the mutual information should be calculated for each gene thoroughly connected to every other gene for correlation [20], the amount of correlation coefficients is tremendous and grows significantly with increasing number of genes. Thus, most publications applied the mutual information algorithms to measure coexpression on public sample datasets or testing datasets that includes much fewer genes than initial datasets.

In order to investigate the expression correlation of immune genes, we constructed a database named MIrExpress (http://wjx.bjfu.edu.cn/MIrExpress) including 41,477 probe sets for 20,283 human genes with each of the 16 cell types in immune cells to reflect the linear and nonlinear correlation of cell-specific gene coexpression profiles across multiple experimental conditions, aided by both Pearson correlation coefficient (*r*) and mutual information value (MI). Through a web interface, the database exhibits the scatter plot of the cooccurrence signal values of any two probe pairs to illustrate the extent and strength of correlation. For a given gene pair, not only is the MI given through the web interface, but its rank expressed in percentage is also presented in all the gene pairs, that is, about 8.6 × 10^8^ pairs for each dataset. Besides, it is the same case for the Pearson correlation value *r*. Both the values and ranks of MI and *r* are displayed and contrasted graphically. In the querying web pages, the top 10 most relevant genes of an input gene can be listed with the perspective of Pearson correlation, mutual information, and their hybrid, respectively.

## 2. Materials and Methods

### 2.1. Data Preparation and Preprocessing

Gene Expression Omnibus (GEO) founded by National Center for Biotechnology Information (NCBI) in July 2000 is the largest public database to date for gene expression data (http://www.ncbi.nlm.nih.gov/geo/) [22]. In this paper, the SOFT format annotation files in GEO database were downloaded from the platform GPL570 for human cells. According to the SOFT files, samples related to immune cells were screened and sorted by cell types. Based on cell-specific sample ID, the raw gene expression data in CEL format files were downloaded from the GEO database using the GEOquery package [23] in R language environment, each expression data containing a single value describing the signal intensity for each probe set on the array.

In order to help improve the efficiency of the data analyzing process, the functions in the packages of Bioconductor were performed on the gene expression data. Firstly, package “simpleaffy” was used to discard the samples with extreme values in order to control the quality of raw data including scale factor, background level, percentage of genes which are called present, and 3′/5′ ratios as the QC metrics [24]. After quality control, 6,909 human samples for 293 GEO series were selected as they were done in Immuco database [13]. Secondly, in the package “affy,” MAS 5.0 algorithms including background correction, normalization, and summary were applied to generally process qualified gene expression data which are allowed for comparison among the gene expression data of samples from different experiments [25, 26]. After that, 41,477 probe sets for human organism were retained for later gene coexpression correlation analysis while about 15% of the samples were discarded due to quality control.

### 2.2. Calculation of Pearson Correlation Coefficient

Pearson correlation coefficient (*r*) is a measure of the linear correlation between two probe sets *X* and *Y*, which can be denoted by *r*_*X*,*Y*_ and calculated as follows:

(1) $$\begin{array}{c} r_{X,Y}=\frac{\sum_{i=1}^{n}\left(X_{i}-\overset{-}{X}\right)\left(Y_{i}-\overset{-}{Y}\right)}{\sqrt{\sum_{i=1}^{n}{\left(X_{i}-\overset{-}{X}\right)}^{2}\sum_{i=1}^{n}{\left(Y_{i}-\overset{-}{Y}\right)}^{2}}}, \end{array}$$

where *n* is the number of samples from different experiments and *X*_*i*_ and *Y*_*i*_ are the expression profiles' values of probe sets *X* and *Y* in the *i*th sample, respectively.

**Figure 1:**
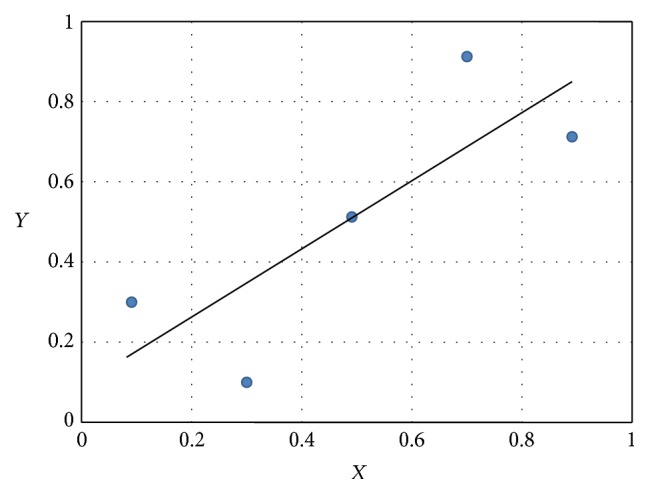
A scatter plot shows expression data of probe sets *X* and *Y* for dataset [(0.1,0.3), (0.3,0.1), (0.5,0.5), (0.7,0.9), (0.9,0.7)].

The *r* values range between −1 and +1, in which 1 implies total positive correlation, −1 total negative correlation, and 0 no correlation between the probe set pairs. The simple example in Figure 1 is a scatter plot about expression data of probe sets *X* and *Y* for a dataset, and the corresponding Pearson correlation coefficient *r* computed using (1) is 0.8. It indicates that there is strongly linear correlation between the probe set pairs.

### 2.3. Statistical Analysis about Mutual Information

The concept of entropy originates in physics, which measures the disorder of a thermodynamic systems. Shannon [27] originally devised the entropy to study the amount of information in a transmitted message and constructed information theory. So far, entropy has wide applications in various fields. Based on the theory of entropy, mutual information is applied to measure the information contained in one probe set about the other. If the mutual information of two probe sets is high, it means that it is easy to predict the expression value of one probe set according to the expression value of the other, which indicates that there may be a close relationship between genes. On the other hand, if the mutual information of two probe sets is zero, it implies that the two variables (two genes) are independent and do not correlated [14]. Based on the entropy theory, we implemented the mutual information approach to study gene coexpression.

According to the concept of mutual information, we regard a probe set as a discrete random variable and calculate the mutual information of two probe sets as the following process [21]. Suppose that *A* is the value range of a probe set *X* and *A* is divided by the subinterval set {*A*_*i*_}, *i* = 1,2,…, *M*, satisfying that ⋃_*i*_{*A*_*i*_} = *A* and that *A*_*i*_∩*A*_*k*_ = *∅* if *i* ≠ *k*. The entropy *H*(*X*) of the probe set *X* can be defined as

(2) $$\begin{array}{c} H\left(\;X\;\right)=-\overset{M}{\underset{i=1}{\sum }}p\left(\;A_{i}\;\right)\log_{2}p\left(\;A_{i}\;\right), \end{array}$$

where probabilities *p*(*A*_*i*_) are approximated by the corresponding relative frequencies of occurrence in *A*_*i*_ and can be calculated as

(3) $$\begin{array}{c} p\left(\;A_{i}\;\right)⟶\frac{k_{i}}{N}, \end{array}$$

where *k*_*i*_ denotes the number of gene expression data in the subsection *A*_*i*_ and *N* is the total number of gene expression data for the probe set *X* [16]. When the probability *p*(*A*_*i*_) is 1 and all other probabilities *p*(*A*_*j*_) with *i* ≠ *j* are zero, we get the minimum of *H*(*X*), zero. In contrast, if *p*(*A*_*i*_) = 1/*M* for each *A*_*i*_, maximum of *H*(*X*) can be reached as log_2_⁡*M*. The joint entropy *H*(*X*, *Y*) of two probe sets *X* and *Y* is defined as

(4) $$\begin{array}{c} H\left(\;X,Y\;\right)=-\overset{M_{X}}{\underset{i=1}{\sum }}\;\overset{M_{Y}}{\underset{j=1}{\sum }}p\left(\;X_{i},Y_{j}\;\right)\log_{2}p\left(\;X_{i},Y_{j}\;\right). \end{array}$$

Here *p*(*X*_*i*_, *Y*_*j*_) denotes the joint probability that *X* is in subinterval set {*A*_*i*_}, *i* = 1,2,…,*M*_*X*_, and *Y* is in subinterval set {*B*_*j*_}, *j* = 1,2,…, *M*_*Y*_, and *p*(*X*_*i*_, *Y*_*j*_) can be computed approximately as

(5) $$\begin{array}{c} p\left(\;X_{i},Y_{j}\;\right)⟶\frac{k_{ij}}{N}. \end{array}$$

In the above equation, *k*_*ij*_ denotes the number of gene expression data when *X* lies in *A*_*X*_ and *Y* in *B*_*Y*_. If the probe sets *X* and *Y* are statistically independent, we can get the joint entropy *H*(*X*, *Y*) after factorizing the joint probabilities as the following formula [16]:

(6) $$\begin{array}{c} H\left(\;X,Y\;\right)=H\left(\;X\;\right)+H\left(\;Y\;\right). \end{array}$$

The mutual information *I*(*X*, *Y*) between the probe sets *X* and *Y* is then defined as

(7) $$\begin{array}{c} I\left(\;X,Y\;\right)=H\left(\;X\;\right)+H\left(\;Y\;\right)-H\left(\;X,Y\;\right)⩾0. \end{array}$$

When the probe sets *X* and *Y* are statistically independent, the mutual information *I*(*X*, *Y*) is zero according to (6) and (7). In sum, *I*(*X*, *Y*) can be taken as measure of correlation no matter whether the correlation is linear or nonlinear. According to (2) and (3), (7) can be rewritten as

(8) IX,Y−∑i=1MXpXilog2⁡pXi−∑j=1MYpYjlog2⁡pYj+∑i=1MX ∑j=1MYpXi,Yjlog2⁡pXi,Yj=log2⁡N+1N∑i=1MX ∑j=1MYkijlog2⁡kijkikj.

We now use the above formula of mutual information to estimate the value about MI for dataset *A* in Figure 1. The dataset consists of *N* = 5 data points divided into *M*_*X*_ = *M*_*Y*_ = 5 bins with fixed intervals and the resulted value of mutual information *I*(*X*, *Y*) computed using (8) is 2.322.

**Figure 2:**
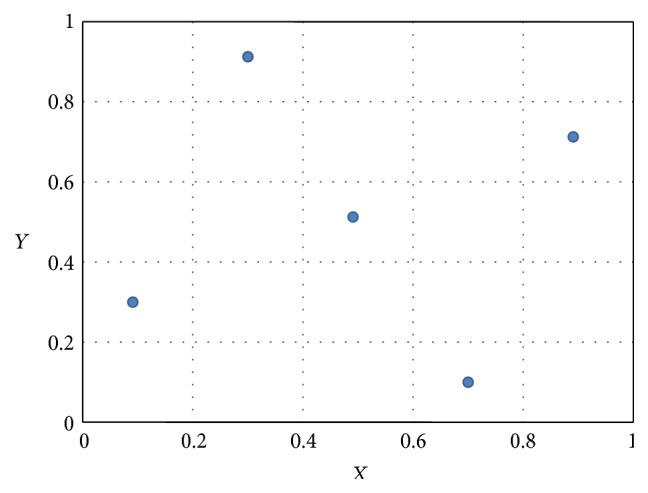
A scatter plot about expression data of probe sets *X* and *Y* with fixed intervals to divide the axes into discrete bins. Dataset = [(0.1,0.3), (0.3,0.9), (0.5,0.5), (0.7,0.1), (0.9,0.7)].

If we change the positions of the data points in Figure 1 and rearrange them to a state as shown in Figure 2, we will find that *Y* is equally dependent on *X* as before, because an occurrence of *X* is equally capable of predicting the occurrence of *Y* as before. That is to say, the MI remains to be 2.322 without any change. The Pearson correlation coefficient *r*, however, changes dramatically from 0.8 to 0, which implies that *X* and *Y* are now not linearly correlated at all. This simple example indicates that MI can generally measure the dependency including both the linear and nonlinear correlation between two probe sets and overcome the drawback of Pearson correlation that takes only the linear correlation into account.

It is a simple approach to estimate the probabilities for gene expression data occurrence in each interval by (3), but it leads to overestimating the mutual information for finite-size datasets [28]. Instead of dividing the expression data range into equal intervals, we adopted an adaptive partitioning strategy to calculate mutual information between two variables (two probe sets here) [16, 29]. It means that the value range of each probe set is divided into *M* discrete nonoverlapping intervals, each containing approximately *N*/*M* data points. The width of each interval is thus various according to the density of data points and more occupied regions are covered with smaller intervals. For instance, let *M* = 11 and the entropy *H*(*X*) and *H*(*Y*) in (2) can be described as *H*(*X*) = *H*(*Y*) = −log_2_⁡11. Consequently, the mutual information *I*(*X*, *Y*) between probe sets *X* and *Y* can be calculated as

(9) $$\begin{array}{c} I\left(\;X,Y\;\right)=2\log_{2}11+\overset{M_{X}}{\underset{i=1}{\sum }}\;\overset{M_{Y}}{\underset{j=1}{\sum }}\frac{k_{ij}}{N}\log_{2}\frac{k_{ij}}{N}. \end{array}$$

## 3. Calculation

For improved measuring effect, the Pearson correlation coefficient and mutual information can be jointly applied to evaluate the strength of gene coexpression. The Pearson correlation coefficient *r* reflects the linear correlation between any two genes, while the mutual information MI generally measures the dependency of one gene on another, both linearly and nonlinearly. But the range and the distribution of values for these two measures are different (MI ∈ [0, +*∞*), *r* ∈ [−1,1]); thus it is not suitable to compare these two measures directly to quantify the linear and nonlinear correlation between any gene pairs [16, 30], and so we adopted the rank ordering of MI and *r* as coexpression measure in the MIrExpress database instead. So we need to compare ordering ranks of MI and *r* instead of their values; that is, we need to find the ranks of MI and *r* in more than 8.6 × 10^8^ values, respectively. However, it is both space-inefficient and time-inefficient to find the rank of a given value in that large amount of values. In fact, ranks in percentage are sufficient because we do not need the rank ordering information more detailed than the percentage.

In order to get the MI rank of any gene pair, all we need now is a vector *V* = {*v*_*i*_}, *i* = 0,1,…, 100, where *v*_0_ is the minimum mutual information and *v*_100_ is the maximum one, and the mutual information of approximately 1 percent gene pairs resides between *v*_*i*−1_ and *v*_*i*_, *i* = 1,2,…, 100. With the vector *V*, we can directly search the proper interval that a given MI resides in and thus find how many mutual information values in percentage are smaller than this MI. It is by this way that we reduce the memory consumption from about *O*(8.6 × 10^8^) to *O*(101).

It is easy for us to save the vector *V*, but difficult to obtain it, for we have no prior knowledge about the distribution of the mutual information values unless we scan the whole pairs and rearrange them. That means we have to record each information value of all the gene pairs for later use, which would consume large amount of memory. Fortunately we noticed that if the expression value range is divided into 11 intervals (as we did with MIrExpress database), the mutual information of any gene pair is between 0 and 3.5, and so we equally divided the MI value range [0,3.5] into 35,000 subintervals and counted the number of pairs whose mutual information resides in a given interval. After scanning all the gene pairs, we obtained another vector with integer values *U* = {*u*_*j*_}, *j* = 1,2,…, 35000, where *u*_*j*_ is the number of gene pairs whose mutual information is in the interval [(*u*_*j*_ − 1)/10000, *u*_*j*_/10000). By using this compression technique, we reduced the memory consumption from more than *O*(8.6 × 10^8^) to *O*(35,000), yet still retaining high accuracy.

Besides the above two techniques of space-saving, we designed highly time-efficient algorithms to accelerate the coexpression analysis. Firstly, the initial values (expression data) were preprocessed and only the corresponding interval information was saved. Thus, the expression of each gene in about 1,000 samples was designated to one of the 11 intervals and the variable values needed in (9) are well-prepared. The second technique was constructing a table for the (*k*_*ij*_/*N*)log_2_⁡(*k*_*ij*_/*N*) part in (9). We noticed that the number of different values of (*k*_*ij*_/*N*)log_2_⁡(*k*_*ij*_/*N*) is no more than the number of samples that is less than 2,000. And so we saved the values in a table *T* indexed by the integer value *k*_*ij*_, and thus we were able to search the table instead of computing the complex function.

With the vectors *V*, *U* and the table *T* well-prepared, it was relatively easy for us to calculate the MI for any given gene pair and its rank in all the MI values. Firstly, the expression value pair for each sample was divided into one element of an 11-by-11 matrix. Then, we could look up all the function values of (*k*_*ij*_/*N*)log_2_⁡(*k*_*ij*_/*N*) in the table *T* and add them up according to (9) to get the MI. Finally we would look up the MI value in the vectors *V* and *U* to get the knowledge of how many MI values are smaller than this one and at which percentage this value is located.

## 4. Results

### 4.1. The Rank of MI and *r*

The MIrExpress database displays a global view of cell-specific gene expression profile across different experiment conditions through two-dimensional scatter plots whose axes represent the signal values of two probe sets. The scatter plot provides database users significant intuition about the general coexpression level of two genes. As is mentioned above, it is not suitable for us to directly compare MI and *r* to find dependency level, linear correlation level, and linear component of the dependency relation, since their value ranges and distributions are widely different. However, it is much more reasonable if we compare their value ranks, that is, where the MI and *r* are located in all sorted MI values and *r* values, respectively. For example, if the rank of an MI is 70%, then it means that 70 percent of all the MI values are smaller than this MI value.

We denote the rank of an MI in all the sorted MI values by RoMI and that of *r* by Ror. In immune cells, there are 4 cases for two given probe sets when we have calculated their RoMI and Ror.

**Figure 3:**
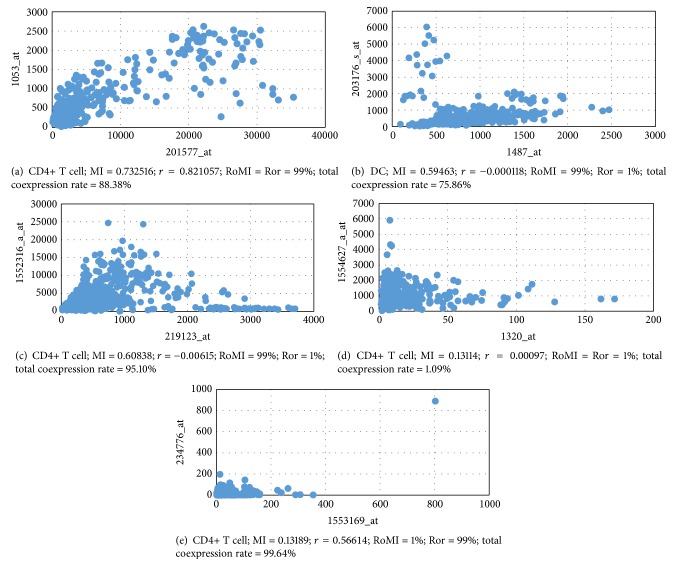
Sample applications for gene coexpression analysis. (a) NME1 and RFC2 in CD4+ T cells. (b) ESRRA and TFAM in DC cells. (c) ZNF232 and GIMAP1 in CD4+ T cells. (d) PTPN21 and ASCC1 in CD4+ T cells. (e) LRRN4 and DMBX1 in CD4+ T cells.

(1) Both RoMI and Ror are high. For example, the MI and *r* of probe sets ID 201577_at (Gene Symbol: NME1) and 1053_at (Gene Symbol: RFC2) in CD4+ T cells are 0.732516 and 0.821057, respectively, and the RoMI and Ror of these two measures are both 99%. This indicates that there exists strong linear and nonlinear correlation and coexpressed relationship between these two probe sets (Figure 3(a)).
(2) The RoMI is high while the Ror is low. For example, there is strongly coexpressed relationship (total coexpression rate = 75.86%) between probe sets 1487_at (Gene Symbol: ESRRA) and 203176_s_at (Gene Symbol: TFAM) in DC cells (dendritic cells), which cannot be reflected through *r* value (−0.000118). If we employ MI value to measure the dependent relationship in MIrExpress database, then the MI is 0.59463 and the corresponding RoMI is 99%, a much higher rank than Ror (1%), which indicates a weak linear correlation but a strong nonlinear correlation between the probe sets (Figure 3(b)). Take CD4+ T cell, for instance, and there is the strong coexpressed relationship (total coexpression rate = 95.10%) between probe sets 219123_at (Gene Symbol: ZNF232) and 1552316_a_at (Gene Symbol: GIMAP1), which cannot be reflected by *r* value (−0.006148), either. But inquiring MIrExpress database, we get the RoMI and Ror as 99% and 1%, respectively (Figure 3(c)). Through these two examples we can observe that mutual information (MI) and the rank of it (RoMI) better interpret the coexpression relationship between probe sets than Pearson correlation coefficient (*r*) and the rank of it (Ror). So mutual information provides a more reliable and reasonable explanation of gene coexpression.
(3) Both RoMI and Ror are low. For example, the MI and *r* of probe sets ID 1320_at (Gene Symbol: PTPN21) and 1554627_a_at (Gene Symbol: ASCC1) in CD4+ T cells are 0.13114 and 0.00097, respectively, and the corresponding RoMI and Ror of these two measures are both 1%. It indicates that both linear and nonlinear correlation are quite weak between these probe sets (Figure 3(d)).
(4) The RoMI is low while the Ror is high. For example, the MI and *r* of probe set ID 1553169_at (Gene Symbol: LRRN4) and 234776_at (Gene Symbol: DMBX1) in CD4+ T cells are 0.13189 and 0.56614, respectively, and the corresponding RoMI and Ror are 1% and 99%, respectively (Figure 3(e)). But we notice that the absent-absent rate (AA) is 99.64% and present-present rate (PP) is 0.00%, which makes it seems true that the two probe sets are strongly linear-correlated. In fact, they are not indeed highly linear-correlated, because a high AA together with a low PP makes the Pearson correlation coefficient quite great. The low RoMI is consistent with the vanishing dependency between the two probe sets who both have low expression level in almost all samples.

### 4.2. Database Contents

**Figure 4:**
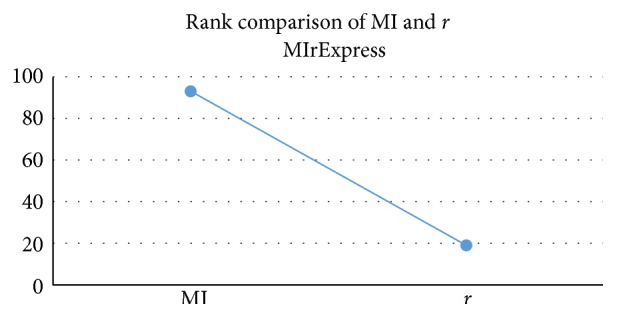
Page for pairwise correlation analysis. The scatter diagram of probe set pair is omitted which appears as Figures 3(a), 3(b), 3(c), 3(d), and 3(e). The species is “human”; the dataset is “CD3+ T cell”; for Gene A, the probe set ID is “1007_s_at” and the gene symbol is “DDR1”; for Gene B, the probe set ID is “1053_at” and the gene symbol is “RFC2.” The Pearson's *r* value is −0.05538, the MI value is 1.04368, and the MIr value is 0.36477 for their hybrid.

We built the MIrExpress database (Browser/Server architecture) adopting Apache Tomcat as web server and MySQL as database server, and it provides users an easy-understanding web interface. All samples for the MIrExpress database are based on immune cells including 16 human cell groups, and the expression data of samples are chosen for Affymetrix Human Genome U133 plus 2.0 Array from GEO database. The web interface of MIrExpress database mainly includes three types of pages: page for pairwise correlation analysis (see Figure 4), page for most related genes, and page for cell-type-based overview of rank difference.

**Figure 5:**
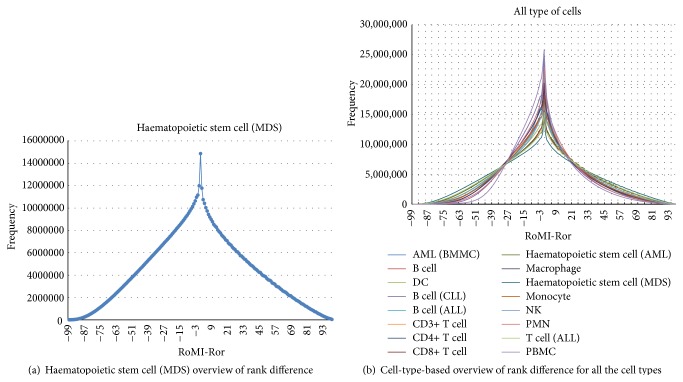
Page for cell-type-based overview of rank difference.

(1) Page for pairwise correlation analysis presents the general expression level of any two genes specified by users among 41,477 probe sets. Users only need to select the cell types and input two queried genes by symbol (e.g., DDR1 and RFC2) or probe sets (e.g., 1007_s_at and 1053_at) in the querying box and click the submitting button to acquire the two-dimensional scatter plot for these two genes. Meanwhile, the MI and *r*, together with the corresponding RoMI and Ror and their comparison, are displayed in the responding page.
(2) Page for most related genes lists information about the 10 most strongly correlated genes to the queried one with 3 perspectives, namely, MI, *r*, and their hybrid, respectively. We use MIr to denote the hybrid measure of MI and *r*, calculated as the follows:
  (10) MIr=βrXi,Xjmaxk≠i⁡rXi,Xk+1−βMIXi,Xjmaxk≠i⁡MIXi,Xk,
 where *X*_*i*_ is the queried gene, *X*_*j*_ is any other one, and *β* is a coefficient often set to be round 0.5 for optimum effect. For example, if a user inputs a probe set 1007_s_at of CD3+ T cell in the selected page and submits the selection, then 30 probe sets and their gene information will be retrieved, in which 10 probe sets are most related to the queried probe set according to the *r*, another 10 to MI, and still another 10 to MIr (Table 1).
(3) The page for cell-type-based overview of rank difference provides for each cell type an overview of how all the RoMI-Ror values are distributed. For instance, the RoMI-Ror value distribution overview of haematopoietic stem cell (MDS) type is shown in Figure 5(a), in which the horizontal axis represents the difference between RoMI and Ror, namely, RoMI-Ror, and the vertical axis represents the frequency of a given difference RoMI-Ror which occurs. And the sum of all the frequency is 860,150,026. Specifically, MI and *r* have the same rank when RoMI-Ror = 0, and we observe that the frequency of this rank difference is the highest. As the difference RoMI-Ror gradually increases (or decreases) from this point, the frequency that the corresponding difference occurs gradually decreases. The rank difference (RoMI-Ror) distributions of all the 16 cell types are shown in Figure 5(b).

### 4.3. Managing and Expanding the Database

The website and the database are totally automatic in responding the users' query if there is no abnormity. However, human operators are required to be involved to expand the database. In fact, in order to increase the visit speed of the website, we have preprocessed all the data before they are mounted into the database, and thus all newly acquired expression data should be preprocessed by human operators with the preprocessing software, individually or in batch.

## 5. Conclusions and Discussions

The MIrExpress database provides an effective and novel method to observe linear and nonlinear dependencies for pairwise gene expression data under a series of experiment conditions in immune cells. To date, this cannot be achieved in other related databases about correlation of gene expression. Traditionally, standard methods, such as Pearson correlation, are used to identify gene coexpression and correlation relationships. However, in some cases, coexpression relationship exists obviously but the Pearson correlation coefficient cannot reflect the dependency, which indicates that there is nonlinear correlation between gene pairs. In this paper, we took into account the rank ordering of mutual information and Pearson correlation coefficient to generally measure the gene correlation in linear and nonlinear aspects, which better describes the gene coexpressions.

There is much room for the MIrExpress database to be improved. First, much more samples may also be incorporated to enrich the database content in order to more precisely measure the correlation in the future. Second, the more kinds of cells, especially those of animals, can be incorporated into a next version of MIrExpress to more extensively reveal coexpression relationship between gene pairs. Third, a pressing need from a variety of applications is to cluster the genes according to mutual information or its variations in order to find interesting gene groups within which the genes share common functional tasks and regulatory mechanisms and thus offer insights into various transcriptional and biological processes.

## Figures and Tables

**Table 1:** Page for most related genes.

The most relevant probe sets to 1007_s_at								
Most relevant probe sets to Gene A (according to MI)			Most relevant probe sets to Gene A (according to *r*)			Most relevant probe sets to Gene A (according to MI*r*)		
Probe set	Gene symbol	Pearson's *r*	Probe set	Gene symbol	Pearson's *r*	Probe set	Gene symbol	Pearson's *r*
225437_s_at	C7orf27	1.26033	223460_at	CAMKK1	0.72291	219071_x_at	C8orf30A	0.76873
203028_s_at	CYBA	1.2656	202182_at	KAT2A	0.73306	1570410_at	CYGB	0.77039
229348_at	UBIAD1	1.26796	40359_at	RASSF7	0.73899	48580_at	CXXC1	0.77492
227811_at	FGD3	1.27493	222674_at	C9orf114	0.74328	40359_at	RASSF7	0.77801
206138_s_at	PI4KB	1.2802	1555866_a_at	HEXDC	0.74495	36545_s_at	SFI1	0.78158
36545_s_at	SFI1	1.28182	43977_at	TMEM161A	0.74878	229348_at	UBIAD1	0.79097
1570410_at	CYGB	1.2902	221629_x_at	C8orf30A	0.75543	43977_at	TMEM161A	0.79156
211512_s_at	OGFR	1.29953	208779_x_at	DDR1	0.75607	213681_at	CYHR1	0.79345
203419_at	MLL4	1.35051	213681_at	CYHR1	0.76656	221629_x_at	C8orf30A	0.80694
210749_x_at	DDR1	1.56079	210749_x_at	DDR1	0.90993	210749_x_at	DDR1	1
